# Maternal Chronic HBV Infection Would Not Increase the Risk of Pregnancy-Induced Hypertension – Results from Pregnancy Cohort in Liuyang Rural China

**DOI:** 10.1371/journal.pone.0114248

**Published:** 2014-12-05

**Authors:** Xin Huang, Hongzhuan Tan, Xun Li, Shujin Zhou, Shi Wu Wen, Meiling Luo

**Affiliations:** 1 Department of Epidemiology and Health Statistics, School of Public Health, Central South University, Changsha, Hunan, China; 2 Liuyang Municipal Hospital of Maternal and Child Health, Beizheng, Liuyang, Hunan, China; 3 OMNI Research Group, Department of Obstetrics and Gynecology, University of Ottawa, Ottawa, Ontario, Canada; CEA, France

## Abstract

The relationship between maternal HBV (hepatitis B virus) infection and pregnancy-induced hypertension (PIH) is inconclusive. Few studies have been conducted in rural areas of China. In order to examine the association between maternal chronic HBV infection and risk of PIH in Liuyang rural area China, we enrolled 6,195 eligible pregnant women in 2010–2011 in selected 14 towns of Liuyang on their first prenatal visit to local maternity care unit. A total of 461 subjects (7.44% (95%CI: 6.79%, 8.10%)) were identified with positive HBsAg status (exposed group) and 5734 were non-HBV carriers (unexposed group). Multivariate log-binomial regression models were used to estimate the risk of PIH, gestational hypertension (GH), and preeclampsia (PE) in relation to maternal chronic HBV infection. There are total of 455 subjects diagnosed with PIH (7.34% (95%CI: 6.70%, 7.99%)), including 371 GH (5.99% (95%CI: 5.40%, 6.58%)) and 81 PE (1.31% (95%CI: 1.07%, 1.64%)). The crude risk ratio between PIH, GH, PE and maternal HBV infection were 1.20 (95%CI: 0.88, 1.64), 1.30(95%CI: 0.93, 1.81) and 0.79 (95%CI: 0.32, 1.93), respectively. After adjustment for gravidity history, abortion history, family history of Diabetes Mellitus (DM) and family history of hypertension, positive HBsAg status was still not significantly associated with PIH (RR = 1.18, 95%CI: 0.87, 1.62), GH (RR = 1.27, 95%CI: 0.91, 1.78) or PE (RR = 0.79, 95%CI: 0.32, 1.95). Additional adjustment for maternal age, marital status, parity history, family history of DM, Body Mass Index at first antenatal visit, folic acid supplementation, smoking status during pregnancy and economic status of living area, multivariate analysis provided similar results. In conclusion, our study found that maternal chronic HBV infection prevalence rate is 7.4% among Liuyang rural area and there is no significant association between maternal HBV infection and the risk of PIH, GH or PE.

## Introduction

More than 400 million people in the worldwide are chronically infected with Hepatitis B virus [1], [2]. The majority of them live in Asia Pacific including China [3]. In China, the prevalence of hepatitis B surface antigen (HBsAg) in individuals aged 1–59 years was 7.2% [4]. Consequently, chronic HBV infection affects a significant number of Chinese pregnant women. Maternal asymptomatic infection with HBV has been strongly associated with increased risk of medical complications during pregnancy, for example, antepartum hemorrhage and gestational diabetes mellitus [5]–[13]. The relationship between chronic HBV infection and risk of pregnancy-induced hypertension (PIH), however, remains unclear.

PIH, which includes gestational hypertension (GH) and preeclampsia (PE), is a major cause of maternal, fetal, and neonatal morbidity and mortality [14], [15]. Furthermore, PIH could increase the risk of long-term cardiovascular diseases of both mothers and their children [16], [17]. PIH became the second leading cause of maternal death in China, which accounted for 13.8% and 8.8% of total maternal deaths in urban and rural areas, respectively [18]. Although significant efforts have been made, little is known about the etiology of PIH.

Early published studies have reported a conflicting association between maternal chronic HBV infection and PIH. Some studies found a significantly positive association between maternal HBV infection and PIH [5], some reported no association between GH or PE and HBV [6]–[12], while others found a significantly negative association between GH, PE and HBV [13], [19], [20]. In addition, previous studies have been done in USA [6], [7], Germany [8], Israel [9], Iran [5], Thailand [10] and Hong Kong [11]–[13], [19], [20]. To our best knowledge, no study in China mainland rural area has been reported. The incidence of PIH varied between different races [21], [22] and the prevalence of HBV infection varied not only between different countries [2], [23], but also within different areas of the same country [24], [25]. The findings from previous studies might not be generalizable to Chinese rural population. Given the inconsistent relationship between chronic HBV infection and PIH and the paucity of studies in China rural area, we conducted a study in Liuyang, Hunan, China, to examine the association between HBV and PIH among China rural population.

## Methods

### Study population

Data for this study were collected within our pre-conception cohort in Liuyang which was designed to study pregnancy induced hypertension and gestational diabetes mellitus. Liuyang is a ‘county-level city’ with 4 city districts and 33 towns. It is located in northeast Hunan Province and under the jurisdiction of Changsha City. Economic status and population size of rural areas in China have wide variation. According to the outcome of Changsha statistical survey [26], multi-stage sampling method was used to select the study subjects. Firstly, based on local GDP level, all 33 towns of Liuyang County were stratified into high, middle and low income areas. Secondly, according to the overall population size ratio of those areas (4∶4∶5), four towns from 10 high income townships, 5 from 12 middle income townships and 5 from 11 low income townships were selected. All pregnant women (6,693) living in those selected 14 towns of Liuyang from January 2010 to December 2011 were asked to participate in the cohort on their first prenatal visit to local maternity care unit. Of them, 6,237 subjects (93%) were followed to childbirth. Furthermore, women who gave multiple births, still birth, or who had chronic hypertension or chronic hepatitis C virus infection were also excluded, which yielded a final sample size of 6,195.

### Data collection and variable definition

Our data collection has consisted of two components: antenatal care booklet collection and hospital chart review. In rural China, all pregnant women were offered with maternal healthcare based on three-level network system (county hospital, the township hospitals and village clinics). Information on antenatal care would be routinely recorded in an antenatal care booklet since first prenatal visit by certified doctors or nurses. This booklet was kept by individual during pregnancy and should be handed in to local maternity care unit in town after childbirth for the process of applying for birth certification. We collected antenatal care booklets of all subjects in township maternity care unit every month. And subjects' hospital medical records were linked to their booklets as long as they give birth at hospitals. General information on maternal demographic characteristics, health status, blood pressure (BP) during pregnancy, obstetric history and antenatal care records could be obtained from antenatal care booklets. Information on maternal complications was abstracted from antenatal care booklets and hospital records.

Maternal hepatitis B surface antigen (HBsAg) status was part of the routine antenatal care in Liuyang and will be routinely reconfirmed when pregnant women were hospitalized for childbirth. HBsAg was screened by enzyme linked immunosorbent assay (ELISA) kit. Maternal chronic infection with HBV was defined as positive HBsAg status on antenatal record. In Liuyang, BP was measured 3 times by certified nurse, using standardized mercuric-column sphygmomanometer at each prenatal visit in a sitting position after 5 minutes of rest, and the time interval between successive pairs of BP measurements was 2 minutes. GH was defined as systolic BP (SBP) ≥140 mmHg and/or diastolic BP (DBP) ≥90 mmHg, occurring for the first time after 20 weeks of gestation. PE was defined as SBP ≥140 mmHg and/or DBP ≥90 mmHg, concurrent with proteinuria (≥0.3 g in a 24-hour urine specimen or ≥1+ on dipstick in two urine samples) after 20 weeks of gestation. PIH was defined as a combination of GH and PE. According to HBsAg status, subjects were classified into HBsAg positive (exposed) and HBsAg negative (unexposed) groups.

### Ethics statement

The study protocol was reviewed and approved by the Central-South University's Ethical and Confidentiality Committee. All participants provided written informed consent.

### Statistical analysis

Chi-square tests were used to compare the distributions of maternal characteristics between exposed and unexposed group. Crude risks of PIH by different maternal characteristics and their 95% confidence intervals (CI) were estimated based on normal approximation method. Multivariate log-binomial regression models were used to calculate risk ratios (RR) and 95%CI for the association between HBV infection and risk of PIH, GH and PE. Potential confounding variables included maternal age (<25, 25–34, and ≥35 years), martial status (unmarried or married), gravidity (≤1,>1), nulliparous (yes or no), abortion history (yes or no), family history of DM (yes or no), family history of hypertension (yes or no), BMI at first antenatal visit (<18.5, 18.5–24.9, and ≥25 kg/m^2^), folic acid supplement intake during pregnancy (yes or no), smoking during pregnancy (yes or no) and economic status of living area (high, middle and low income area). Additional adjustment for alcohol consumption, infant gender, weight gain during pregnancy did not result in material changes of the observed associations and thus were not included into the final models (results not shown). Statistical significance was assessed at the 5% level (two-tail test). All analyses were performed using SAS software, version 9.2 (SAS Institute, Inc., Cary, NC).

## Results

Of the 6,195 study subjects, 461 (7.44% (95%CI: 6.79%, 8.10%)) subjects were with positive HBsAg status (Table 1). Compared to unexposed group, HBsAg positive carriers were more likely to be multigravida, having abortion history and family history of hypertension. There were no significant differences in the distribution of maternal age, marital status, parity history, family history of DM, BMI at first antenatal visit, taking folic acid supplement during pregnancy, exposure to active smoking and economic status of living area between the exposed and unexposed groups.

**Table 1:** Distribution of selected characteristics of subjects between maternal HBV carriers and non-HBV carriers, Liuyang, Hunan, China, 2012.

Characteristics		HBsAg (−)		HBsAg (+)		χ^2^	*P*
		N = 5734	%	N = 461	%		
Age (years)							
	<25	3520	61.39	282	61.17	0.032	0.984
	25–34	2072	36.14	167	36.23		
	≥35	142	2.48	12	2.60		
Marital status							
	Unmarried	186	3.24	11	2.39	1.020	0.313
	Married	5548	96.76	450	97.61		
Gravidity							
	≤1	2492	43.46	176	38.18	4.966	0.026
	>1	3234	56.40	285	61.82		
Nulliparous							
	No	1758	30.66	149	32.32	0.553	0.457
	Yes	3976	69.34	312	67.68		
Abortion history							
	No	3928	68.50	281	60.95	11.165	0.001
	Yes	1806	31.50	180	39.05		
Family history of DM							
	No	5719	99.74	458	99.35	2.231	0.135
	Yes	15	0.26	3	0.65		
Family history of hypertension							
	No	5662	98.74	449	97.40	5.791	0.016
	Yes	72	1.26	12	2.60		
BMI at first antenatal visit (kg/m^2^)							
	<18.5	1253	21.85	85	18.44	3.020	0.221
	18.5–24.9	3861	67.34	322	69.85		
	≥25	620	10.81	54	11.71		
Folic acid supplement							
	No	1743	30.40	145	31.45	0.225	0.636
	Yes	3991	69.60	316	68.55		
Smoking							
	No	5686	99.16	459	99.57	0.867	0.352
	Yes	48	0.84	2	0.43		
Economic status of living area							
	High income	1664	29.0	130	28.2	0.157	0.924
	Middle income	1780	31.0	146	31.7		
	Low income	2290	39.9	185	40.1		

Abbreviations: HBsAg, hepatitis B virus antigen; BMI, body mass index; DM, diabetes mellitus.

There are total of 455 subjects diagnosed with PIH (7.34% (95%CI: 6.70%, 7.99%)), including 371 GH (5.99% (95%CI: 5.40%, 6.58%)) and 81 PE (1.31% (95%CI: 1.07%, 1.64%)) (Table 2). Crude risks of PIH by other different maternal characteristics were also showed in Table 2. Those with age over 35, family history of hypertension, BMI at first antenatal visit over 25 or not taking folic acid supplement during pregnancy were having higher risks of PIH than others.

**Table 2:** Crude risks of pregnancy-induced hypertension by different maternal characteristics among pregnant women in Liuyang rural area, Hunan, China, 2012.

Characterist-ics		Observed population	GH/PE	GH	PE
		N	Risk(%)(95%CI)	Risk(%)(95%CI)	Risk(%)(95%CI)
Overall		6195	7.34(6.70, 7.99)	5.99(5.40, 6.58)	1.36(1.07, 1.64)
Age (years)					
	<25	3802	5.76(5.02, 6.50)	4.81(4.13, 5.49)	0.95(0.64, 1.25)
	25–34	2239	8.71(7.54, 9.88)	6.97(5.91, 8.02)	1.74(1.20, 2.28)
	≥35	154	26.62(19.64, 33.60)	20.78(14.37, 27.19)	5.84(2.14, 9.55)
Marital status					
	Unmarried	197	6.60(3.13, 10.07)	5.08(2.01, 8.14)	1.52(0.00, 3.23)
	Married	5998	7.37(6.71, 8.03)	6.02(5.42, 6.62)	1.35(1.06, 1.64)
Gravidity					
	≤1	2668	7.27(6.29, 8.26)	5.96(5.06, 6.86)	1.31(0.88, 1.74)
	>1	3527	7.40(6.54, 8.26)	6.01(5.23, 6.80)	1.39(1.00, 1.78)
Nulliparous					
	No	1907	7.18(6.03, 8.34)	5.82(4.77, 6.87)	1.36(0.84, 1.88)
	Yes	4288	7.42(6.63, 8.20)	6.06(5.35, 6.78)	1.35(1.01, 1.70)
Abortion history					
	No	4209	7.44(6.64, 8.23)	5.94(5.23, 6.65)	1.50(1.13, 1.86)
	Yes	1986	7.15(6.02, 8.28)	6.09(5.04, 7.14)	1.06(0.61, 1.51)
Family history of DM					
	No	6177	7.32(6.67, 7.97)	5.97(5.38, 6.56)	1.34(1.06, 1.63)
	Yes	18	16.67(0.00, 33.88)	11.11(0.00, 25.63)	5.56(0.00,16.14)
Family history of hypertension					
	No	6111	7.22(6.57, 7.87)	5.87(5.29, 6.46)	1.34(1.05, 1.63)
	Yes	84	16.67(8.70, 24.64)	14.29(6.80, 21.77)	2.38(0.00, 5.64)
BMI at first antenatal visit (kg/m^2^)					
	<18.5	1338	5.68(4.44, 6.92)	4.86(3.71, 6.01)	0.82(0.34, 1.31)
	18.5–24.9	4183	6.67(5.91, 7.43)	5.36(4.67, 6.04)	1.31(0.97, 1.66)
	≥25	674	14.84(12.15, 17.75)	12.17(9.70, 14.63)	2.67(1.45, 3.89)
Folic acid supplement					
	No	1888	9.22(7.91, 10.52)	7.84(6.63, 9.05)	1.38(0.85, 1.90)
	Yes	4307	6.52(5.79, 7.26)	5.18(4.52, 5.84)	1.35(1.00, 1.69)
Smoking					
	No	6145	7.32(6.67, 7.97)	5.97(5.38, 6.56)	1.35(1.06, 1.64)
	Yes	50	10.00(1.68, 18.32)	8.00(0.48, 15.52)	2.00(0.00, 5.88)
Economic status of living area					
	High income	1794	7.02(5.84, 8.21)	5.91(4.82, 7.00)	1.28(0.08, 1.80)
	Middle income	1926	7.63(6.45, 8.82)	5.54(4.55, 6.54)	1.40(0.09, 1.93)
	Low income	2475	7.35(6.33, 8.38)	5.79(4.89, 6.68)	1.37(0.09, 1.83)

Abbreviations: BMI, body mass index; DM, diabetes mellitus; CI, confidence intervals.

The crude risks of PIH and GH among positive HBsAg carrier were 8.68% and 7.59% respectively, which is higher than that unexposed group (7.24% and 5.86%) (Table 3). Meanwhile, the risk of PE among positive HBsAg carrier was 1.08% lower than that unexposed group (1.38%). However, none of the risks difference between those two groups were statistical significant. After adjustment for gravidity history, abortion history, family history of DM and family history of hypertension, positive HBsAg status was still not significantly associated with PIH (RR = 1.18, 95%CI: 0.87, 1.62), GH (RR = 1.27, 95%CI: 0.91, 1.78) or PE (RR = 0.79, 95%CI: 0.32, 1.95). Additional adjustment for maternal age, marital status, parity history, family history of DM, BMI at first antenatal visit, folic acid supplementation, smoking and economic status of living area, multivariate analysis provided similar results (Table 3).

**Table 3:** Association between maternal HBV infection and pregnancy-induced hypertension, Liuyang, China, 2012.

	HBsAg +		HBsAg -		Crude RR (95%CI)	Adjusted RR^a^ (95%CI)	Adjusted RR^b^ (95%CI)
	N = 461	Risk(%) (95%CI)	N = 5734	Risk(%) (95%CI)			
GH/PE	40	8.68 (6.10, 11.26)	415	7.24 (6.57, 7.91)	1.20 (0.88, 1.64)	1.18 (0.87, 1.62)	1.17(0.85, 1.59)
GH	35	7.59 (5.17, 10.02)	336	5.86 (5.25, 6.47)	1.30 (0.93, 1.81)	1.27 (0.91, 1.78)	1.25(0.90, 1.76)
PE	5	1.08 (0.13, 2.03)	79	1.38 (1.08, 1.68)	0.79 (0.32, 1.93)	0.79 (0.32, 1.95)	0.80(0.32, 1.96)

a Adjustment covariates are gravidity, abortion history, family history of hypertension.

b Adjustment covariates are maternal age, marital status, gravidity, parity, abortion history, family history of DM, family history of hypertension, BMI at first antenatal visit, folic acid supplementation, smoking, economic status of living area.

Abbreviations: GH, gestational hypertension; PE, preeclampsia; HBsAg, hepatitis B virus antigen; RR, risk ratio; CI, confidence intervals.

Only 5 subjects (1% of HBsAg positive carriers) suffered with chronic active HBV infection, others were chronic carriers with normal liver function. We also tried to exclude those chronic active infection subjects. This sensitive analysis reached the same conclusion (data not shown).

## Discussion

Our population based on cohort study shows that maternal chronic HBV infection prevalence rate is 7.4% (95%CI: 6.79%, 8.10%) among Liuyang rural area. This prevalence rate is similar to the overall population of China 7.2% (95%CI: 6.67%, 7.70%) [4] and those women of childbearing age (15–49) in Jiangsu, China (6.71% (95%CI: 6.10%, 7.30%)) [27] or in Northwest China (7.2% (95%CI: 6.0%, 8.5%)) [24]. But it is lower than women of childbearing age in Hainan, China (9.5% (95%CI: 8.99%, 10.02%)) [28] and pregnant women in Hong Kong(10.0% (95%CI: 9.78%, 10.18%)) [19]. According to the study in China, the rate of mother-to-child transmission of HBV was 7.3% [29]. Therefore, the high maternal HBV infection rate in our study area would remain a serious concern. Timely administration of the HBV vaccine combined with hepatitis B immune globulin need to be administered to at risk individuals [30].

There are 11 previously published studies that had assessed the association between maternal HBV infection and risk of PIH and reached inconsistent conclusion. Of those 11 studies, 2 were multi-center based [6], [7] and 9 were single hospital based [5], [8]–[13], [19], [20]. Those 2 multi-center based studies were conducted in US and observed the same results as our population based cohort study. One was conducted by Reddick et al. [6], they used the discharge registry data from 1054 hospitals across 37 states, enrolled 297,664 subjects with 814 HBV carriers. After adjustment for maternal age, race and other confounding variables, no association was observed between maternal HBV infection and PE (OR = 0.91, 95%CI: 0.57, 1.4). The other one was conducted in Florida [7], using births certification and hospital discharge linked data and enrolled 1, 670, 369 subjects with 1,458 HBV carriers, also found no significant association for HBV and GH or PE. Studies done by Sirilert et al. in Thailand [10], Lobstein et al. in Germany [8], Safir et al. in Israel [9], Wong et al. [11] and Tse et al. [12] in Hong Kong also support this conclusion. However, a case-control study in Iran [5], enrolled 450 HBV carriers and 450 controls observed increased risk of PIH with maternal HBV infection (OR = 4.2, 95%CI: 2.2, 8.1). In contrast to all of the aforementioned studies, Lao et al. [19] enrolled 86,537 subjects with 8,634 HBV carriers in a teaching hospital in Hong Kong found that maternal HBV infection could reduce the risk of PIH (OR = 0.79, 95%CI: 0.66, 0.95) and PE (OR = 0.71, 95%CI: 0.56, 0.91). And other two studies in Hong Kong [13], [20] also supported this negative association. All of those single hospital based studies were conducted in hospitals with great annual delivery rate. The potential patients served by those hospitals may differ from those served by regular community hospitals. In another words, the admission rate of non-HBV carriers with PIH in those hospitals may differ from that of HBV carriers with PIH. We speculated that this discrepancy might involve selection bias and result in the inconsistence results.

Previous studies have demonstrated that HBV infection could increase the risk of atherosclerosis [31], however this association was secondary to the liver dysfunction associated with HBV infection rather than HBsAg positivity itself [32], though HBV infection could induce a proinflammatory effect. 99% of our study population was chronic HBV carrier with normal liver function. The simple HBV infection without liver dysfunction may not initiate or aggravate vascular damage and causes atherosclerosis, which were the key the process of PE. The association between maternal chronic active HBV infection and PIH may need further investigation.

Our study has several strengths in comparison with previous studies of the field. It was the population-based prospective cohort study with reduced selection bias. The data contained detailed information on maternal demographic and medical and some pregnancy information allowing adjustment for several important potential confounding factors simultaneously. Diagnosis of GH/PE in our study was based on medical records not self-report, which minimized potential disease misclassification. However, the limitation of our study should be considered when interpreting the study findings. Our data did not include information on HBcAg, HBeAg or HBV DNA levels. Therefore, we were not able to determine whether the replicating virus had an effect on onset of PIH. Only 5 subjects suffered chronic active HBV infection, this limitation did not allow us to do further research on the association between chronic active HBV infection and PIH. Lack of information on the date of diagnosis of PIH limited our ability to examine the association by early and later onset PE, which might have different etiologies. The incidence of PE and PIH and prevalence of HBV infection among Chinese population are quite different from that of Caucasians, genetic differences may play a dominant role. Hence, the generalizability of our results may be limited to Chinese population.

## Conclusions

Our study found that maternal chronic HBV infection prevalence rate is 7.4% among Liuyang rural area and there is no significant association between maternal HBV infection and the risk of PIH, GH or PE.
